# Hematologic Responses to Molidustat in Cats with Anemia of Heterogeneous Etiology: A Retrospective Descriptive Study

**DOI:** 10.3390/vetsci13101075

**Published:** 2026-10-11

**Authors:** Gippeum Lee, Jeongmin Lee, Kun-Ho Song

**Affiliations:** 1 Department of Veterinary Internal Medicine, College of Veterinary Medicine, Chungnam National University, Daejeon 34134, Republic of Korea; vetmania@cnu.ac.kr (G.L.); memo7677@cnu.ac.kr (J.L.); 2 Korea Animal Medical Center, Cheongju 28651, Republic of Korea

**Keywords:** anemia, cat, chronic kidney disease, darbepoetin, hypoxia-inducible factor prolyl hydroxylase inhibitor, molidustat, retrospective study

## Abstract

Molidustat, an oral hypoxia-inducible factor prolyl hydroxylase inhibitor, is approved for nonregenerative anemia associated with chronic kidney disease (CKD) in cats but is also used for anemia of other causes. This retrospective, single-center study described the hematologic response to molidustat in 39 cats with anemia of heterogeneous etiology and identified factors that complicate attribution of improvement to the drug. Response was defined as a hematocrit (HCT) increase of ≥4 percentage points from baseline at the complete blood count closest to day 28 (days 14–45), without transfusion to treat anemia. Molidustat was used outside the approved indication in 33 cats (84.6%). Seventeen cats (43.6%) had a response-window measurement; they had a longer treatment span and differed in etiology and concurrent treatment from non-evaluable cats. Among these 17 cats, 12 (70.6%; 95% CI 46.9–86.7%) met the response criterion; sensitivity analyses specified before formal analysis gave 70.6–75.0% and post hoc attribution analyses 29.4–50.0%. In 7 of the 12 responders, darbepoetin or transfusion preceded the qualifying measurement, or it was obtained more than 7 days after the last dose. These data describe clinical use but do not establish effectiveness outside CKD-associated anemia.

## Simple Summary

Molidustat is an oral medicine approved for anemia caused by chronic kidney disease in cats. Veterinarians sometimes also use it for anemia with other causes, but little is known about what happens in these cats. We reviewed the records of 39 anemic cats that received molidustat at one hospital. Most had anemia that was not caused by kidney disease alone, and many also received other treatments, such as blood transfusions or another medicine that stimulates red blood cell production. Only 17 cats had a complete blood count (CBC) within the predefined response window, and 12 of these met the predefined response criterion (a rise in hematocrit, the percentage of the blood made up of red blood cells). However, in more than half of these 12 cats, other treatments or the timing of the blood test meant that attribution of the improvement to molidustat was uncertain. Carefully designed prospective studies are needed before molidustat can be recommended for anemia other than that caused by kidney disease.

## 1. Introduction

Nonregenerative anemia is a common complication of chronic kidney disease (CKD) in cats, becoming more prevalent as disease progresses through International Renal Interest Society (IRIS) stages 3 and 4, and contributes to lethargy, inappetence and reduced quality of life [1]. Current guidelines recommend active management of anemia in cats with CKD [2,3], with treatment indicated when the hematocrit (HCT) or packed cell volume (PCV) is <25% or persistently 25–28% [2].

Erythropoiesis-stimulating agents (ESAs), recombinant human erythropoietin and, more recently, darbepoetin (DPO) [4] have been used off-label for decades to manage CKD-associated anemia in cats. However, ESA therapy has recognized drawbacks, including anti-erythropoietin antibody formation and pure red cell aplasia in an estimated 25–30% of cats treated with human recombinant erythropoietin (less common with darbepoetin), iron deficiency, hypertension and polycythemia [1,5], in addition to the need for injection. Hyporesponsiveness to ESAs occurs in 20–40% of human patients with CKD, often because of functional iron deficiency associated with chronic inflammation [6].

Molidustat is a hypoxia-inducible factor prolyl hydroxylase inhibitor (HIF-PHI). By inhibiting the enzymes that degrade HIF-α, it stabilizes HIF and increases endogenous erythropoietin production, and it may also improve iron availability through suppression of hepcidin [6,7]. It is available as an oral, once-daily formulation (Varenzin-CA1). Erythropoietic activity was first demonstrated in healthy laboratory cats [8], then in a small placebo-controlled pilot study in client-owned cats with CKD-associated anemia [9], and subsequently in a multicenter, randomized, double-masked, placebo-controlled trial of 75 client-owned cats, in which 68% of molidustat-treated cats and 17% of placebo-treated cats met the treatment-success criterion on day 28, defined as an HCT increase of ≥4 percentage points or a ≥25% relative increase from baseline, or both [10]. On this basis, molidustat received conditional approval from the US Food and Drug Administration for the control of nonregenerative anemia associated with CKD in cats [11], and it has since been authorized in Great Britain and the European Union for nonregenerative anemia associated with CKD in cats [12,13].

The published evidence derives from cats meeting trial eligibility criteria, namely cats with CKD-associated anemia (PCV < 28%) that had not previously received ESAs, blood transfusion or anabolic steroids and did not have severe inflammation unrelated to CKD [10]. In clinical practice, anemia in cats frequently has more than one cause: in large retrospective series, CKD accounted for less than half of cases, and inflammatory, neoplastic and multifactorial causes accounted for much of the remainder [14,15,16]. Anemic cats are also often treated with transfusion, ESAs, iron supplementation or corticosteroids, and medicines not authorized for cats are commonly used in feline practice [17]. Whether a hematologic response follows molidustat administration in cats with anemia of other causes, or in cats receiving concurrent treatments, has not been systematically described.

The objective of this study was to describe, in a retrospective single-center cohort, the hematologic response to molidustat across the anemia etiologies encountered in clinical practice, and to identify factors, particularly concurrent ESA therapy and acute interventions, that complicate attribution of any observed improvement to molidustat. The study was not designed to establish efficacy; its purpose was to characterize response patterns in clinical use and to generate hypotheses for future prospective studies.

## 2. Materials and Methods

### 2.1. Study Design, Population and Eligibility

This retrospective, single-center, descriptive and exploratory cohort study, reported in accordance with applicable items of the STROBE-Vet extension for observational studies in veterinary medicine [18], was conducted at Korea Animal Medical Center, Cheongju, Republic of Korea. Its aim was to describe hematologic changes in cats with anemia of heterogeneous etiology that received molidustat (Varenzin-CA1; Elanco Animal Health), an oral HIF-PHI, in routine clinical practice. The study was not designed to establish efficacy or safety.

During the study period, molidustat was not licensed as a veterinary medicinal product in the Republic of Korea and was, therefore, not available through an authorized domestic distribution channel. Varenzin-CA1 was a veterinary medicinal product conditionally approved by the US Food and Drug Administration for the control of nonregenerative anemia associated with CKD in cats [11]. In the context of limited therapeutic options, the product used in this cohort was obtained overseas by the study hospital for the treatment of individual client-owned cats under the attending veterinarian’s clinical judgment and responsibility. Before treatment, owners were informed that molidustat was not licensed as a veterinary medicinal product in the Republic of Korea, and consent was obtained as described in the Informed Consent Statement. The present study is a retrospective analysis of the records generated during this clinical care and was not a prospective clinical trial designed to establish the efficacy or safety of molidustat. The available stock was depleted in late March 2025. Because stock was limited, prescriptions were allocated preferentially to newly treated cats and were often dispensed in short courses. Treatment span and continuation were, therefore, influenced by drug availability as well as by clinical response.

**Figure 1:**
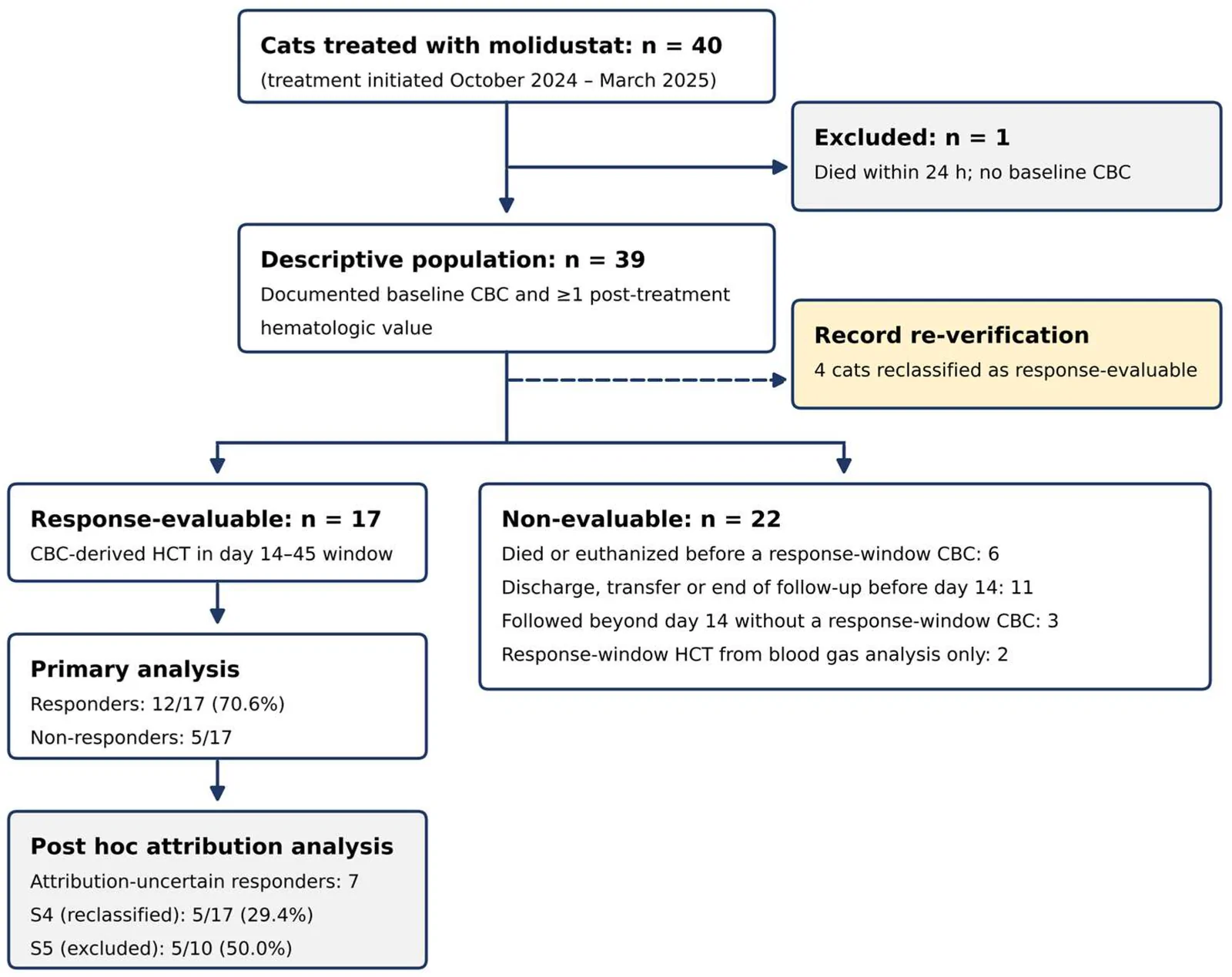
Flow of cats from identification to the descriptive and response-evaluable populations, with reasons for non-evaluability. Solid arrows indicate the flow of cats between groups; the dashed arrow indicates record re-verification, after which four cats were reclassified as response-evaluable. CBC, complete blood count; HCT, hematocrit.

Cats that received at least one dose of molidustat for anemia with treatment initiated between October 2024 and March 2025 were eligible; follow-up data, including survival status, were collected until 15 August 2026 (data lock). Anemia was diagnosed by the attending veterinarian in the context of the underlying disease; no predefined numeric threshold was applied at the time of treatment. In three cats with baseline HCT ≥ 30% (Section 3.2), all with CKD and admitted for RRT, molidustat was started in anticipation of anemia during RRT rather than for anemia documented at baseline. For descriptive purposes, the lower limit of the in-house analyzer reference interval (HCT 30%) was used as a reference value, and the baseline HCT distribution is reported relative to this value and to guideline treatment thresholds (HCT < 25%, or persistently 25–28%) [2]. No cats were excluded on the basis of etiology, comorbidity, concurrent treatment or previous treatment; the only cat excluded was one that died within 24 h of admission before a baseline CBC could be obtained (Figure 1).

Two analysis populations were defined. The descriptive population comprised all cats with a documented baseline CBC and at least one post-treatment hematologic value. The response-evaluable population comprised cats in the descriptive population with a CBC-derived HCT within the response window (Section 2.4). Cohort flow from identification to each analysis population, with reasons for non-evaluability, is shown in Figure 1.

For all cats, evaluability was re-verified against the original medical records (clinical notes, treatment and billing records, and laboratory reports) after initial data extraction. This re-verification identified response-window CBCs that had been omitted during initial extraction in four cats, which were reclassified as response-evaluable. Three cats in this cohort (one response-evaluable and two non-evaluable) were included in a previously published case series describing hematologic and renal trends in cats undergoing renal replacement therapy with molidustat use [19]; their data are included here as part of the full cohort. No a priori sample size calculation was performed; the cohort comprised all eligible cats during the study period.

### 2.2. Etiologic Classification, Regenerative Status and Indication Status

Anemia etiology was classified into one of seven categories: CKD-associated, inflammatory/chronic disease-associated, neoplasia-associated, blood loss-associated, critical illness-associated, multifactorial, and other/undetermined [14,15,16,20]. The inflammatory/chronic disease-associated category comprised anemia attributed to infectious, inflammatory or other chronic systemic disease; cats with immune-mediated anemia were not included in this category. Immune-mediated anemia was diagnosed or presumptively diagnosed on the basis of bone marrow examination, clinical findings (e.g., splenomegaly) or response to immunosuppressive therapy. Because each such cat also had another contributing condition (e.g., FeLV infection), these cats were classified as multifactorial, and the immune-mediated component and its diagnostic basis were recorded. Operational criteria for each category are listed in Table S1. Where more than one diagnosis plausibly contributed and no single cause predominated, the case was classified as multifactorial and the contributing diagnoses were recorded.

CKD was diagnosed according to IRIS criteria [21]. Because several cats presented with acute-on-chronic kidney disease, creatinine-based stage at presentation is reported descriptively and is not used as a formal chronic-phase stage.

Regenerative status at baseline was classified from the absolute reticulocyte count on the baseline CBC, with anemia classified as regenerative when the absolute reticulocyte count exceeded 50 × 10^3^/µL [15]. Reticulocyte counts were obtained with the automated analyzer, which does not report aggregate and punctate reticulocytes separately and has not, to our knowledge, been validated against manual aggregate counts in cats; regenerative status is, therefore, reported descriptively [22]. No manual reticulocyte counts on blood smears were performed. The three cats with immune-mediated anemia were tested by PCR for anemia-associated infectious agents, including hemotropic mycoplasmas, to exclude or confirm an infectious cause, and none was positive for hemotropic mycoplasmas. The other cats, including cats with CKD, were not routinely tested for hemotropic mycoplasmas. Screening for FeLV, FIV and heartworm with the SNAP Feline Triple Test (IDEXX) is part of the routine work-up at our hospital. Cats without a baseline absolute reticulocyte count were recorded as unknown.

Indication status was defined relative to the approved indication of molidustat (control of nonregenerative anemia associated with CKD in cats) [11]: cats with CKD-associated nonregenerative anemia were classified as within the approved indication, and all other cats as outside it.

### 2.3. Data Collection, Treatments and Key Variables

Data were extracted from the electronic medical record (e-Friends 365; Woorien Co., Ltd., Hwaseong, Republic of Korea). CBCs were performed with an in-house automated hematology analyzer (Mindray BC-60 Vet; Shenzhen Mindray Bio-Medical Electronics Co., Ltd., Shenzhen, China). Qualifying CBCs for all response-evaluable cats were performed in house. Two cats in the descriptive population, one of them response-evaluable, had a baseline CBC performed at a referring clinic, which used a different analyzer, entered from the referring clinic’s report (see baseline, below).

*Baseline.* Baseline values (HCT, Hb, reticulocyte percentage and absolute count) were taken from the CBC obtained on the day of molidustat initiation or, if unavailable, the most recent CBC within the preceding 7 days. In two cats (one of them response-evaluable), the baseline CBC had been performed at the referring clinic. When two CBCs were obtained on the day of initiation, the value obtained before any transfusion or other acute intervention was used. Where a transfusion or other acute intervention preceded the baseline CBC, the last pre-intervention CBC value was also recorded for sensitivity analysis (Section 2.5). In one cat, the baseline CBC indices were internally inconsistent (MCHC 65.0 g/dL), and its baseline HCT, Hb and reticulocyte values were treated as missing.

*HCT source.* Baseline and response HCT values were required to be CBC-derived. HCT values from point-of-care blood gas analysis were recorded but not used for baseline or response classification.

*Molidustat.* Under the institutional protocol, molidustat was prescribed at 5 mg/kg orally once daily, typically for 21 days and for up to 28 days; the regimen was adjusted according to clinical condition, underlying disease, HCT response and compliance with oral medication. Prescribed dose, frequency, dispensing dates and days supplied were recorded for each cat, and treatment start and last-dose dates were derived from these records and confirmed against clinical notes. For take-home prescriptions, dosing was taken to start on the day after dispensing unless otherwise documented. Treatment span was calculated from the first to the last dose, inclusive, and, therefore, includes any interruptions; the number of days on which molidustat was actually administered was not analyzed separately. Courses exceeding 28 days, repeat courses and treatment interruptions were recorded.

*Darbepoetin.* Under the institutional protocol, darbepoetin alfa was administered at 1 µg/kg subcutaneously once weekly. Administration dates were recorded for each cat. Concurrent darbepoetin exposure was defined as at least one dose administered after molidustat initiation and before the CBC used for response classification; darbepoetin given after that CBC was recorded but not classified as concurrent exposure.

*Transfusion.* Whole-blood transfusions were recorded with date and volume and classified as either associated with extracorporeal renal replacement therapy (RRT; intermittent hemodialysis or prolonged intermittent renal replacement therapy [PIRRT]; administered in anticipation of extracorporeal circuit-related anemia, with prior owner consent) or given to treat anemia.

*Other covariates.* RRT sessions, corticosteroids and other immunosuppressive or antineoplastic drugs, iron supplementation, and other interventions potentially affecting erythropoiesis (e.g., stem cell therapy) were recorded with dates relative to molidustat initiation. Concurrent medications were not controlled by protocol and were recorded as covariates. Iron status (serum iron, ferritin) was not routinely assessed. Iron was prescribed as oral ferric hydroxide twice daily in all 9 cats; the interval between iron and molidustat administration was not systematically recorded.

### 2.4. Hematologic Response Definition and Evaluability

Day 0 was the day of the first molidustat dose. The response window was days 14–45, and the qualifying value was the CBC-derived HCT closest to day 28 within this window. A cat was classified as a responder if HCT at the qualifying CBC had increased by ≥4 percentage points from baseline (the treatment-success criterion reported in the placebo-controlled pilot trial of molidustat in cats with CKD [9], and the absolute component of the criterion used in the pivotal trial, which also accepted a ≥25% relative increase [10]) and no transfusion given to treat anemia had been administered within the response window before the qualifying CBC; otherwise, it was classified as a non-responder. The response threshold was adopted from these trials during protocol development and was finalized before the formal analysis. Because the data had been recorded during routine care, hematologic outcomes existed before the definition was finalized; the robustness of results to the threshold was, therefore, examined in a post hoc sensitivity analysis (Section 2.5).

The original definition did not specify how to handle two CBCs equidistant from day 28. For this revision, the earlier of two equidistant values was used in the primary analysis, and the later value was examined in a sensitivity analysis; this rule was specified after data review and applied to one cat.

Cats without a CBC-derived HCT within the response window were classified as non-evaluable and described separately rather than as non-responders. For each non-evaluable cat, the reason was recorded from the medical record (death or euthanasia before a response-window CBC; discharge, transfer or end of follow-up before day 14; continued follow-up without a response-window CBC; or response-window HCT available only from blood gas analysis). Responder proportions refer only to response-evaluable cats and do not estimate the probability of response among all treated cats.

### 2.5. Statistical Analysis

All analyses were descriptive or exploratory. Categorical variables are reported as *n* (%) and continuous variables as median (interquartile range; range), with the number of cats with missing data for each variable. Responder proportions are reported with 95% confidence intervals (Wilson score method). Baseline HCT and age were summarized descriptively by responder status without hypothesis testing.

Response-evaluable and non-evaluable cats were compared descriptively (baseline characteristics, etiology, CKD status, treatment span, concurrent treatments and reasons for non-evaluability); no hypothesis tests were performed for this comparison.

Exploratory comparisons of responder status by concurrent darbepoetin exposure, CKD status and corticosteroid use are presented as 2 × 2 cell counts with conditional maximum-likelihood odds ratios, exact 95% confidence intervals and Fisher’s exact test *p* values. Change in HCT across etiologic categories was compared with the Kruskal–Wallis test, restricted to categories with at least two cats. Given the small sample, these analyses were not powered to detect associations, and estimates are interpreted as imprecise and hypothesis-generating. No adjustment for confounding and no imputation of missing data were performed.

*Sensitivity analyses specified before formal analysis.* (S1) Exclusion of cats receiving RRT at molidustat initiation. The analysis plan also listed large-volume transfusion at initiation; because the whole-blood volume available per transfusion in cats is limited by single-donor collection (approximately 40–45 mL), a volume-based definition could not be applied, and this component was not implemented as planned (see S1b). (S2) Redefinition of baseline as the last CBC value before any transfusion or other acute intervention.

*Post hoc analyses.* The following were added after case-level review and are reported as post hoc: (S1b) exclusion of cats receiving RRT or a whole-blood transfusion between day −2 and day 2; (S3) use of the later of two CBCs equidistant from day 28; (S6) response thresholds of 3 and 5 percentage points; (S4, S5) attribution analyses in which responders meeting the following attribution-uncertainty criterion were reclassified as non-responders (S4) or excluded (S5). A responder was classified as attribution-uncertain if (A) the qualifying CBC was obtained more than 7 days after the last molidustat dose, or (B) darbepoetin, a transfusion given to treat anemia, or another intervention expected to affect erythropoiesis (e.g., stem cell therapy) was administered after molidustat initiation and before the qualifying CBC. The 7-day threshold was a pragmatic post hoc choice (one week) and was not derived from pharmacokinetic or pharmacodynamic data in cats. Classification under criterion (A) was unchanged for any threshold between 3 and 8 days; with thresholds of 9 days or longer, one or more responders would no longer meet criterion (A). Analyses specified before formal analysis and post hoc analyses are presented together without designating any as the preferred estimate.

Analyses were performed in Python 3.12 (SciPy 1.17). Generative AI (Claude Sonnet 5.5, Anthropic) ) was used to assist with verification of extracted data against source records and with consistency checking of reported statistical and numerical data; all outputs were checked by the authors against the source data.

## 3. Results

### 3.1. Study Population

Forty cats in which molidustat was initiated between October 2024 and March 2025 were identified (Figure 1). One cat died within 24 h of admission before a baseline CBC could be obtained and was excluded from all analyses. The remaining 39 cats formed the descriptive population.

Of the 39 cats, 17 (43.6%) had a CBC-derived HCT within the day 14–45 response window and were response-evaluable, and 22 (56.4%) were non-evaluable. Re-verification of evaluability against the original medical records identified response-window CBCs that had been omitted during initial data extraction in four cats, which were reclassified as response-evaluable. Reasons for non-evaluability were death or euthanasia before a response-window CBC (*n* = 6); discharge, transfer or end of follow-up before day 14 (*n* = 11); continued follow-up beyond day 14 without a response-window CBC (*n* = 3); and a response-window HCT available only from blood gas analysis (*n* = 2) (Figure 1). In one of the three cats followed beyond day 14, molidustat was discontinued after HCT increased to 30% on day 6.

### 3.2. Baseline Characteristics, Etiology and Treatment Exposure

Baseline characteristics of the descriptive population and of the response-evaluable and non-evaluable subgroups are summarized in Table 1, Table 2 and Table 3. Most cats were castrated males (27/39, 69.2%); median age was 10.0 years (IQR 6.5–12.0; range 1–18); and 21/39 (53.8%) had CKD. A valid baseline HCT was available for 38 cats; in one cat, the baseline CBC was not used because its indices were internally inconsistent (MCHC 65.0 g/dL). Median baseline HCT was 21.7% (IQR 18.0–26.0; range 9.6–39.9). Relative to the reference values described in Section 2.1, baseline HCT was <25% in 25/38 cats (65.8%), 25% to <30% in 10/38 (26.3%; 7 of these within 25–28%, persistence not assessable), and ≥30% in 3/38 (7.9%; 31.7%, 38.6% and 39.9%). All three were cats with CKD that received RRT, and molidustat was started in anticipation of anemia during RRT (Section 2.1); in all three, the baseline CBC was obtained immediately before RRT and whole-blood transfusion.

Etiology was classified as multifactorial in 15/39 cats (38.5%), inflammatory/chronic disease-associated in 9 (23.1%), CKD-associated in 7 (17.9%), blood loss-associated in 3 (7.7%), neoplasia-associated in 2 (5.1%), critical illness-associated in 2 (5.1%) and other/undetermined in 1 (2.6%) (Table 4). Immune-mediated anemia was diagnosed or presumptively diagnosed in three cats, on the basis of bone marrow examination, clinical findings or response to immunosuppressive therapy; all three had another contributing condition and were classified as multifactorial (Section 2.2).

A baseline absolute reticulocyte count was available for 33/39 cats (median 16.9 × 10^3^/µL; IQR 8.3–46.5; range 2.3–182.3). Using the threshold in Section 2.2, 7 cats were classified as regenerative and 26 as nonregenerative; regenerative status was unknown in 6. Further, 6 cats (15.4%) had CKD-associated nonregenerative anemia and were classified as within the approved indication; the remaining 33 (84.6%), including 1 cat with CKD-associated anemia that was regenerative at baseline, were outside it (Table 1 and Table 2).

Molidustat was prescribed at 5 mg/kg in all 39 cats and once daily in 38; in 1 cat, dosing was changed to every other day. Median treatment span (first to last dose, including any interruptions) was 9 days (IQR 4.5–22; range 1–163). Eight cats received molidustat over a period longer than 28 days: continuously in two (68 and 29 days), with one or more interruptions in four (including two separate courses in one), as repeated short prescriptions in one, and with a change from once-daily to every-other-day dosing in one.

Darbepoetin was recorded at any time during the study period in 22/39 cats (56.4%); its timing relative to molidustat was verified against the medical record for response-evaluable cats (Table 5). Corticosteroids were recorded in 13/39 (33.3%), iron supplementation in 9/39 (23.1%) and whole-blood transfusion at any time in 16/39 (41.0%). Four cats (10.3%) received RRT at molidustat initiation.

### 3.3. Comparison of Response-Evaluable and Non-Evaluable Cats

Response-evaluable and non-evaluable cats differed in several characteristics (Table 1, Table 2, Table 3 and Table 4). Median molidustat treatment span was 22 days (IQR 14–48) in response-evaluable cats and 5.5 days (IQR 3.2–9) in non-evaluable cats. CKD was recorded in 11/17 (64.7%) response-evaluable and 10/22 (45.5%) non-evaluable cats, and inflammatory/chronic disease-associated anemia in 1/17 (5.9%) and 8/22 (36.4%), respectively; no response-evaluable cat had blood loss-associated or critical illness-associated anemia. Darbepoetin at any time (13/17, 76.5% vs. 9/22, 40.9%) and corticosteroids (8/17, 47.1% vs. 5/22, 22.7%) were recorded more often in response-evaluable cats, and median baseline HCT was 22.3% and 20.3%, respectively. No hypothesis tests were performed for these comparisons.

Response-evaluable cats were, therefore, those that remained under treatment and follow-up for longer, and they differed from non-evaluable cats in etiology and concurrent treatment. The responder proportions reported below describe this subgroup only and do not estimate the proportion of all treated cats that would meet the response criterion.

### 3.4. Hematologic Response Among Response-Evaluable Cats

**Figure 2:**
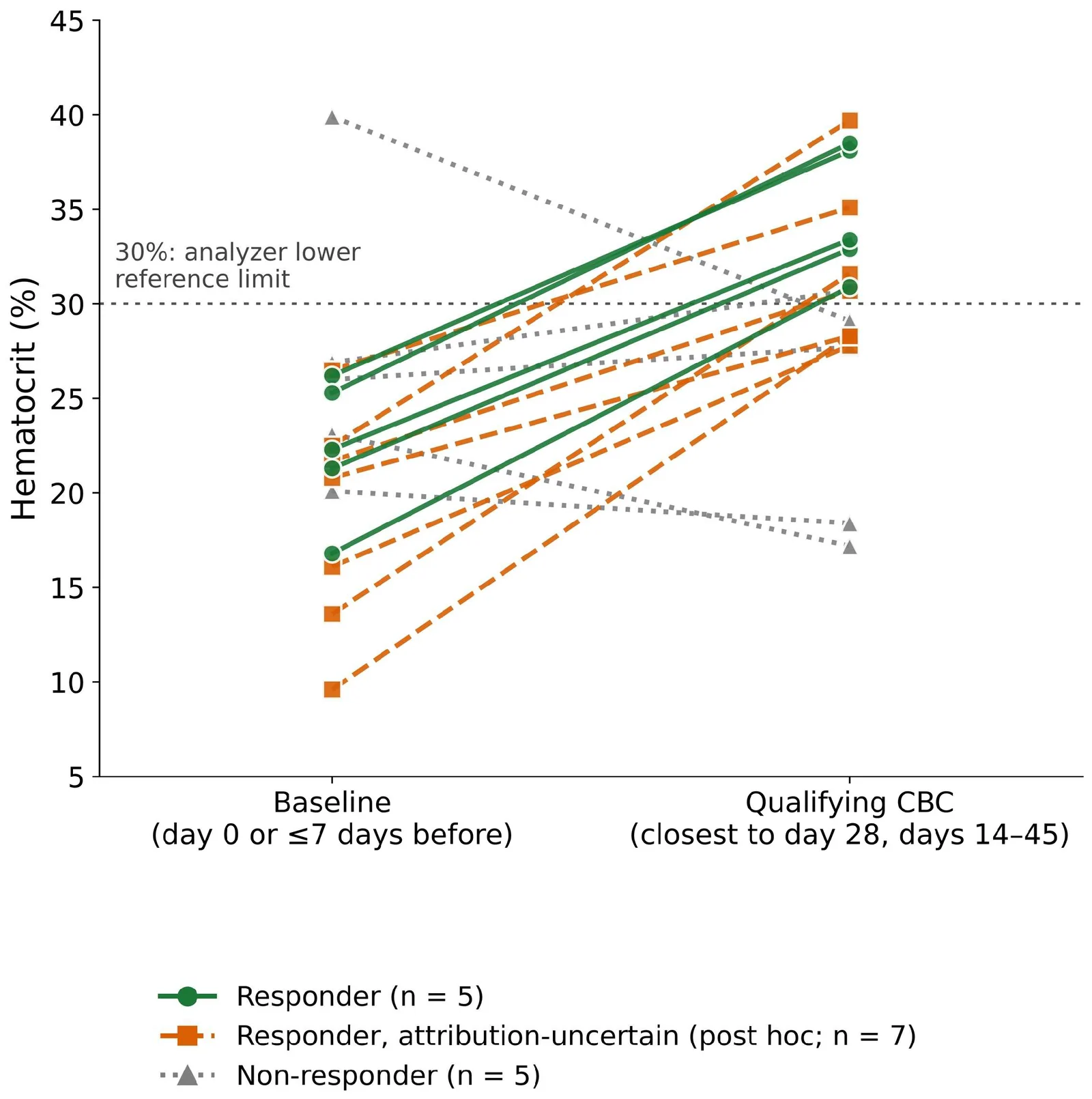
Hematocrit at baseline and at the qualifying CBC in the 17 response-evaluable cats. Each line represents one cat. Solid lines, responders; dashed lines, responders meeting the post hoc attribution-uncertainty criterion; dotted lines, non-responders. The horizontal dotted line indicates the lower limit of the in-house analyzer reference interval (HCT 30%). Individual values are given in Table 5.

Individual data for the 17 response-evaluable cats are shown in Table 5 and Figure 2. The qualifying CBC was obtained at a median of day 27 (IQR 22–30; range 16–36). The median change in HCT from baseline was +11.1 percentage points (IQR +3.8 to +13.2; range −10.8 to +18.7). In the primary analysis, 12 of 17 response-evaluable cats (70.6%; 95% CI 46.9–86.7%) met the response criterion. By etiology, the criterion was met by 4/5 cats with CKD-associated, 6/9 with multifactorial, 1/2 with neoplasia-associated and 1/1 with inflammatory/chronic disease-associated anemia; these subgroup counts are reported descriptively. Of the five non-responders, one had also received a transfusion to treat anemia within the response window. Median baseline HCT was 21.5% (IQR 16.6–23.2; range 9.6–26.5) in responders and 26.0% (IQR 23.1–26.9; range 20.1–39.9) in non-responders, and median age was 10.5 years (IQR 6.8–14.8; range 2–18) and 10 years (IQR 9–16; range 8–17), respectively; these were not compared statistically.

Results of the sensitivity analyses are shown in Table 6. In the sensitivity analyses specified before formal analysis, excluding the one response-evaluable cat that received RRT at molidustat initiation (a non-responder) gave 12/16 (75.0%; 95% CI 50.5–89.8%) (S1). Redefining the baseline as the last value before a transfusion changed the baseline in three cats, all of which remained responders (ΔHCT +17.4, +10.1 and +20.2 percentage points), and the proportion was unchanged at 12/17 (S2).

In the post hoc analyses, additionally excluding cats that received a whole-blood transfusion between day −2 and day 2 gave 8/12 (66.7%; 95% CI 39.1–86.2%) (S1b). Using the later of the two CBCs equidistant from day 28 for the one affected cat (+14.3 percentage points on day 34, compared with +1.7 on day 22) gave 13/17 (76.5%; 95% CI 52.7–90.4%) (S3). Seven of the twelve responders met the attribution-uncertainty criterion: two under criterion (A) alone (qualifying CBC 9 and 24 days after the last molidustat dose) and five under both criteria (A) and (B) (qualifying CBC 13–22 days after the last dose, with darbepoetin administered after molidustat initiation and before the qualifying CBC in all five and a transfusion to treat anemia in one). In the remaining five responders, the qualifying CBC was obtained during treatment or within 2 days of the last dose, and none had received darbepoetin before the qualifying CBC. Reclassifying the seven attribution-uncertain responders as non-responders gave 5/17 (29.4%; 95% CI 13.3–53.1%) (S4), and excluding them gave 5/10 (50.0%; 95% CI 23.7–76.3%) (S5). With response thresholds of 3 and 5 percentage points, the criterion was met by 13/17 (76.5%) and 12/17 (70.6%) cats, respectively (S6).

### 3.5. Exploratory Comparisons

Exploratory comparisons among the 17 response-evaluable cats are shown in Table 7; no data were missing for the variables compared. Darbepoetin was administered after molidustat initiation and before the qualifying CBC in 7 cats, of which 5 (71.4%) met the response criterion, compared with 7 of 10 (70.0%) without such exposure (OR 1.07; 95% CI 0.08–17.40; *p* = 1.00). The criterion was met by 7/11 cats with CKD and 5/6 without (OR 0.37; 95% CI 0.01–5.52; *p* = 0.60), and by 6/8 cats receiving corticosteroids before the qualifying CBC and 6/9 not receiving them (OR 1.46; 95% CI 0.12–23.76; *p* = 1.00). Changes in HCT did not differ significantly across the CKD-associated (*n* = 5; median +11.9 percentage points), multifactorial (*n* = 9; +9.0) and neoplasia-associated (*n* = 2; +5.7) categories (Kruskal–Wallis *H* = 1.30, *p* = 0.52); the single cat with inflammatory/chronic disease-associated anemia was not included in this test. No statistically significant association was detected in any comparison; the estimates were imprecise and do not provide evidence for or against an association.

### 3.6. Case Descriptions

The following cats are described individually because concurrent interventions, treatment interruptions or the timing of the qualifying CBC affect interpretation. Days are relative to molidustat initiation (day 0).

**Case 1** (CKD-associated; acute-on-chronic kidney disease with concurrent hepatobiliary and pancreatic disease) was admitted with severe dehydration and uremia for renal replacement therapy and began molidustat on the day of the first RRT session (PIRRT), in anticipation of anemia during RRT. Baseline HCT (39.9%) and absolute reticulocyte count (102.2 × 10^3^/µL) were measured at presentation, before RRT and transfusion; HCT was within the analyzer reference interval. Darbepoetin was first administered on day 0. Three RRT sessions were performed (days 0, 2 and 6), each accompanied by an RRT-associated whole-blood transfusion (40, 40 and 50 mL); a fourth transfusion (37–40 mL) was given on day 14 to treat anemia. Stem cell therapy was given on day 25. HCT at the qualifying CBC (day 27) was 29.1% (−10.8 percentage points). Molidustat was given intermittently, with interruptions, over a 163-day period. The cat died approximately 15 months after molidustat initiation. The hematologic and renal course of this cat during renal replacement therapy has been reported previously [19].

**Case 4** (CKD-associated; acute-on-chronic kidney disease with ureteral obstruction) received molidustat on days 0–13. Bilateral subcutaneous ureteral bypass devices were placed on day 4, and home subcutaneous fluid therapy, started before referral, was continued. After molidustat stock was depleted, darbepoetin was started on day 14. HCT at the qualifying CBC (day 35), 22 days after the last molidustat dose, was 39.7% (+17.2 percentage points).

**Case 5** (multifactorial) received molidustat from day 0 to approximately day 20. CBCs on day 22 (HCT 27.7%; +1.7 percentage points) and day 34 (40.3%; +14.3) were equidistant from day 28; the earlier value was used in the primary analysis (non-responder) and the later value in S3 (responder). A human placental extract preparation (Laennec) was injected on day 22 after the CBC, the first darbepoetin dose was given on day 29, and the first stem cell treatment was given on day 34 before that day’s CBC. The cat was euthanized on day 68 after acute decompensation of acute-on-chronic kidney disease.

**Case 7** (multifactorial) received molidustat on days 0–8 and darbepoetin on days 0, 8 and 16. HCT at the qualifying CBC (day 30), 22 days after the last molidustat dose, was 27.8% (+11.7 percentage points). The cat was euthanized on day 36.

**Case 11** (multifactorial; suspected precursor-targeted immune-mediated anemia, based on bone marrow examination showing marked erythroid hyperplasia with a left shift [myeloid/erythroid ratio approximately 1:10] despite severe nonregenerative anemia; saline agglutination test negative) received a whole-blood transfusion (50 mL) on day −1, before the baseline CBC (HCT 13.6%; pre-transfusion 14.2%). Molidustat was given on days 0–13, darbepoetin on days 0 and 7, and corticosteroid therapy was ongoing. After discharge to the referring clinic on day 14, the cat continued immunomodulatory therapy and received no transfusion or darbepoetin through day 26. HCT was 26.4% on day 14, 27.1% on day 19 and 31.6% on day 26 (qualifying CBC; +18.0 percentage points; 13 days after the last molidustat dose). HCT subsequently decreased, and on readmission on day 60, the corticosteroid dose was increased and mycophenolate mofetil was started.

**Case 13** (neoplasia-associated; splenic mast cell tumor treated by splenectomy, with suspected gastrointestinal and cutaneous metastasis; receiving toceranib and prednisolone) received darbepoetin on days 5 and 13. Molidustat was given until day 19, and the qualifying CBC (day 33) was obtained 14 days after the last dose (HCT 28.3%; +7.5 percentage points). Baseline absolute reticulocyte count was 59.5 × 10^3^/µL.

**Case 14** (multifactorial; FeLV infection with suspected immune-mediated hemolytic anemia, Coombs test negative) received corticosteroids and a whole-blood transfusion on day −2, before the baseline CBC (HCT 22.3%; pre-transfusion 23.3%). Molidustat was given in two courses: days 0–8 and a 2-week course starting on day 32. The qualifying CBC (day 36; HCT 33.4%, +11.1 percentage points) was obtained during the second course, 28 days after the end of the first course; this cat did not meet the attribution-uncertainty criterion because the qualifying CBC was obtained during treatment. Darbepoetin was first given after the response window.

**Case 15** (multifactorial; mucosal small cell T-cell gastrointestinal lymphoma, enteropathy-associated T-cell lymphoma type II, treated with prednisolone and chlorambucil, and hyperthyroidism treated with methimazole) received molidustat on days 0–12. HCT at the qualifying CBC (day 21), 9 days after the last dose, was 35.1% (+8.6 percentage points). Molidustat was not re-prescribed, and HCT was 30% on day 49.

**Case 17** (multifactorial; severe nonregenerative anemia managed as immune-mediated disease; bone marrow examination showed severe multifocal to coalescing pyogranulomatous to neutrophilic osteomyelitis with fibrosis, periodic acid–Schiff stain negative, and a hemolytic component was also suspected) had a baseline HCT of 9.6% (absolute reticulocyte count 8.3 × 10^3^/µL) before a whole-blood transfusion on day 0. Molidustat was given on days 0 and 1 only. Darbepoetin was administered on day 0, a second transfusion (54 mL) was given on day 2, and immunosuppressive therapy was started on day 1 (dexamethasone on days 1–7, followed by prednisolone and cyclosporine from day 8). HCT was 22.7% on day 6, 25.2% on day 7 (absolute reticulocyte count 96.8 × 10^3^/µL), and 28.3% on day 16 (qualifying CBC; +18.7 percentage points; 15 days after the last molidustat dose). Follow-up at our hospital ended on day 16, after which the cat was managed by the referring clinic.

## 4. Discussion

### 4.1. Principal Findings

This study aimed to describe the hematologic response to molidustat across the anemia etiologies encountered in clinical practice and to identify factors that complicate the attribution of observed improvement to molidustat. In this cohort, molidustat was prescribed across seven etiologic categories, mostly outside the approved indication, and frequently alongside darbepoetin, corticosteroids, iron supplementation and transfusion. Among the 17 response-evaluable cats, 12 (70.6%; 95% CI 46.9–86.7%) met the response criterion in the primary analysis. The sensitivity analyses specified before formal analysis gave similar proportions (70.6–75.0%), whereas the post hoc analyses that addressed attribution gave lower proportions (29.4% when attribution-uncertain responders were counted as non-responders and 50.0% when they were excluded). The wide confidence intervals and the spread across analyses indicate that the observed hematologic improvement cannot be quantified precisely from these data and that, in a substantial proportion of responders, it cannot be attributed to molidustat. The proportion meeting the criterion was similar, with response thresholds of 3 and 5 percentage points (13/17 and 12/17), which were examined because the data had been recorded before the definition was finalized.

Two features of the data limit interpretation of these proportions. First, they describe only the 17 of 39 cats (43.6%) that had a CBC within the response window. Compared with non-evaluable cats, response-evaluable cats had a longer treatment span (median 22 vs. 5.5 days), more often had CKD and received darbepoetin, and less often had inflammatory/chronic disease-associated anemia. Missing follow-up was, therefore, unlikely to be random, and the proportions should not be read as the probability of response among all treated cats. Treatment span was also irregular, because the available stock was depleted in late March 2025 and prescriptions were often dispensed in short courses, in addition to clinical course and owner compliance. Second, 7 of the 12 responders met the post hoc attribution-uncertainty criterion, most often because darbepoetin had been given before the qualifying CBC or because the qualifying CBC was obtained more than 7 days after the last molidustat dose.

### 4.2. Use Outside the Approved Indication

Molidustat was used outside the approved indication in 33 of 39 cats (84.6%), including cats with inflammatory/chronic disease-associated, blood loss-associated, neoplasia-associated and critical illness-associated anemia and one cat with CKD whose anemia was regenerative at baseline. Medicines not authorized for cats are commonly used in feline practice [17], and this cohort documents how molidustat was extended beyond its approved indication at one hospital. HCT increases meeting the response criterion were observed in some cats outside the approved indication, including cats without CKD. However, each etiologic category contained 1–9 response-evaluable cats, no statistically significant difference in HCT change between categories was detected, and several of these cats received concurrent immunosuppressive therapy, darbepoetin or transfusion. These observations do not establish whether molidustat is effective in anemia of other causes; they are reported to describe current practice and to inform the design of future studies. Regenerative status was determined with an automated analyzer that does not distinguish aggregate from punctate reticulocytes and has not, to our knowledge, been validated against manual counts in cats [22]; it was unknown in six cats, and the classification of cats as within or outside the approved indication depends on it. The three cats with immune-mediated anemia tested negative for hemotropic mycoplasmas via PCR, but the other cats were not routinely tested, so interference by circulating organisms with automated reticulocyte counts cannot be excluded in those cats.

### 4.3. Comparison with Published Molidustat Trials

In the pivotal randomized, placebo-controlled trial in cats with CKD-associated anemia, 68% of molidustat-treated cats and 17% of placebo-treated cats met the treatment-success criterion on day 28 [10]. That trial differed from the present cohort in design, population, treatment exposure and outcome assessment (Table 8). It enrolled only cats with CKD-associated anemia (PCV < 28%); excluded cats that had previously received ESAs, blood transfusion or anabolic steroids and cats with severe inflammation unrelated to CKD; administered molidustat for 28 consecutive days with scheduled weekly hematologic monitoring; and defined treatment success on day 28 as an HCT increase of ≥4 percentage points or of ≥25%, or both [10]. Applying this combined criterion post hoc to the present cohort would not have changed the classification of any cat. In the present cohort, treatment span varied with drug availability and clinical course, CBCs were obtained at clinical discretion, and response was assessed at the CBC closest to day 28 within a 14–45-day window. The qualifying CBC was obtained between days 16 and 36. Baseline values were taken from a CBC within 7 days before initiation, a pragmatic limit; in the three cats with baseline HCT ≥ 30%, baseline preceded RRT and transfusion, and in one cat, baseline values were unusable. Because of these differences, the responder proportions of the two studies are not directly comparable, and the present data should not be interpreted as indicating higher or lower effectiveness of molidustat in clinical practice than in the trial.

### 4.4. Concurrent Treatments and Attribution of Response

With respect to the second aim, concurrent treatments were the main factor limiting attribution of hematologic improvement to molidustat. Darbepoetin was administered after molidustat initiation and before the qualifying CBC in 7 of the 17 response-evaluable cats and in 5 of the 7 attribution-uncertain responders. In the exploratory comparison, the proportion meeting the response criterion was similar with and without such exposure (5/7 vs. 7/10); the estimate was imprecise (OR 1.07; 95% CI 0.08–17.40) and does not provide evidence for or against an association between darbepoetin exposure and response in this cohort. Other interventions that may affect erythropoiesis were also common, including transfusion, immunosuppressive therapy in cats with immune-mediated anemia, stem cell therapy and treatment of the underlying disease (Section 3.6). Iron status was not routinely assessed, so its contribution to non-response could not be evaluated [7]. Phosphate binders and iron supplements can chelate HIF-PH inhibitors and reduce their absorption, and the product label advises considering staggered administration of molidustat and these products (at least 1 h apart), if possible [23]; the timing of these products relative to molidustat was not systematically recorded, and phosphate binders were not recorded as a covariate. In one cat with presumptive immune-mediated anemia (Case 17), for example, molidustat was given for 2 days, and HCT recovered during immunosuppressive therapy. Conversely, in one cat (Case 14), the qualifying CBC was obtained during a second treatment course, 28 days after the end of the first, so that the criterion based on time since the last dose did not capture the interruption. These case-level observations show that hematologic change after molidustat initiation in clinical practice may reflect several concurrent processes that the present design cannot separate. The attribution criteria were defined after case review and are reported as post hoc; they are intended to show how sensitive the responder proportion is to these processes, not to identify cats in which molidustat was or was not effective.

### 4.5. Limitations

This was a retrospective, single-center study without a control group, and treatment was allocated by clinical judgment, so confounding by indication cannot be excluded and all estimates are descriptive. The response-evaluable subgroup was small and selected, as discussed above. Safety was not an objective of this study; adverse events, blood pressure and biochemical changes were not systematically recorded or analyzed, and the safety of molidustat outside the approved indication cannot be inferred from these data. Reported adverse events and safety concerns for molidustat include vomiting, increased systolic blood pressure, transient increases in serum potassium, seizures and thromboembolic disease [23]. Concurrent medications, including iron and corticosteroids, were not controlled by protocol, and treatment span varied between cats (once-daily dosing in 38 of 39 cats). Data were extracted retrospectively by one investigator; evaluability was re-verified against the original medical records, which identified omitted response-window CBCs in four cats, but residual extraction errors cannot be excluded. Because molidustat was obtained outside a licensed domestic supply chain, the provenance and storage conditions of the product before receipt could not be independently verified. Finally, three cats in this cohort (one response-evaluable and two non-evaluable) were included in a previously published case series that described their hematologic and renal trends during renal replacement therapy with molidustat use [19]; the present study includes them in the cohort description and, for the response-evaluable cat, applies the response definition described in Section 2.4.

### 4.6. Implications for Practice and Research

When molidustat is used outside its approved indication, the present data support informing owners of the limited evidence, monitoring HCT serially, and recognizing that concurrent darbepoetin, transfusion, immunosuppressive therapy or treatment of the underlying disease may contribute to any observed change. Prospective studies are needed to determine whether molidustat has an erythropoietic effect in anemia other than CKD-associated anemia. Such studies should define eligible etiologies in advance, exclude or stratify by concurrent erythropoiesis-stimulating agents, schedule hematologic monitoring at fixed time points, assess iron status and regenerative status with validated methods, and be sized to estimate response proportions within etiologic groups. Such studies should also record adverse events (including vomiting), blood pressure, serum potassium and other biochemical variables, and clinical status at scheduled time points, none of which was systematically collected in the present cohort. The present cohort can serve as a descriptive basis for the design of such studies.

## 5. Conclusions

This study described the hematologic response to molidustat in cats with anemia of heterogeneous etiology treated in routine practice. In this single-center retrospective cohort, molidustat was used mostly outside its approved indication and frequently together with darbepoetin, transfusion or other treatments that may affect erythropoiesis. Among the 17 of 39 cats with a CBC in the response window, 12 met the response criterion; this proportion applies only to the evaluable subgroup, was imprecise, and decreased when responders with uncertain attribution were reclassified or excluded. Attribution was complicated mainly by concurrent darbepoetin, transfusion and immunosuppressive therapy, by short or interrupted treatment courses, and by incomplete follow-up. These data do not establish the effectiveness of molidustat in anemia other than CKD-associated anemia. They indicate the need for prospective studies with defined populations, scheduled hematologic monitoring and control of concurrent erythropoiesis-stimulating treatment. Until such data are available, the use of molidustat outside its approved indication warrants informed owner consent and serial HCT monitoring.

## Figures and Tables

**Table 1:** Demographic and clinical characteristics of the descriptive population and of response-evaluable and non-evaluable cats. Values are median (IQR; range) or *n* (%).

Characteristic	All (*n* = 39)	Response-Evaluable (*n* = 17)	Non-Evaluable (*n* = 22)
Age, years	10.0 (6.5–12.0; 1–18)	10.0 (8.0–16.0; 2–18)	8.0 (5.2–11.8; 1–17)
Body weight, kg	4.0 (3.0–5.2; 1.9–8.5)	4.3 (3.4–5.2; 2.9–6.3)	3.9 (2.7–4.8; 1.9–8.5)
Sex, CM/SF/M	27 (69.2)/11 (28.2)/1 (2.6)	15 (88.2)/2 (11.8)/0	12 (54.5)/9 (40.9)/1 (4.5)
CKD present	21 (53.8)	11 (64.7)	10 (45.5)
Within/outside the approved indication	6 (15.4)/33 (84.6)	4 (23.5)/13 (76.5)	2 (9.1)/20 (90.9)

CM, castrated male; SF, spayed female; M, intact male; CKD, chronic kidney disease.

**Table 2:** Baseline hematologic values of the descriptive population and of response-evaluable and non-evaluable cats. Values are median (IQR; range) or *n* (%); *n* with data shown where fewer than the column total.

Characteristic	All (*n* = 39)	Response-Evaluable (*n* = 17)	Non-Evaluable (*n* = 22)
Baseline HCT, %	21.7 (18.0–26.0; 9.6–39.9) [*n* = 38]	22.3 (20.1–26.0; 9.6–39.9)	20.3 (18.0–25.9; 10.8–38.6) [*n* = 21]
HCT < 25%/25 to <30%/≥30%	25/10/3 [*n* = 38]	11/5/1	14/5/2 [*n* = 21]
Baseline Hb, g/dL	7.3 (6.0–9.3; 2.6–13.9) [*n* = 38]	8.0 (6.1–9.3; 2.6–11.8)	6.9 (6.0–9.3; 4.0–13.9) [*n* = 21]
Baseline absolute reticulocytes, ×10^3^/µL	16.9 (8.3–46.5; 2.3–182.3) [*n* = 33]	13.0 (10.1–29.3; 3.8–102.2) [*n* = 15]	27.8 (8.2–53.0; 2.3–182.3) [*n* = 18]
Regenerative/nonregenerative/unknown	7/26/6	2/13/2	5/13/4

HCT, hematocrit; Hb, hemoglobin. Baseline HCT, Hb and reticulocyte values of one non-evaluable cat were treated as missing because the CBC indices were internally inconsistent.

**Table 3:** Treatment exposure of the descriptive population and of response-evaluable and non-evaluable cats. Values are median (IQR; range) or *n* (%).

Characteristic	All (*n* = 39)	Response-Evaluable (*n* = 17)	Non-Evaluable (*n* = 22)
Molidustat treatment span, days	9 (4.5–22; 1–163)	22 (14–48; 2–163)	5.5 (3.2–9; 1–38)
Darbepoetin, any time	22 (56.4)	13 (76.5)	9 (40.9)
Corticosteroids	13 (33.3)	8 (47.1)	5 (22.7)
Iron supplementation	9 (23.1)	5 (29.4)	4 (18.2)
Whole-blood transfusion, any time	16 (41.0)	5 (29.4)	11 (50.0)
RRT at molidustat initiation	4 (10.3)	1 (5.9)	3 (13.6)

RRT, extracorporeal renal replacement therapy (intermittent hemodialysis or prolonged intermittent renal replacement therapy). Timing of darbepoetin and transfusion was verified against the medical record for response-evaluable cats only.

**Table 4:** Anemia etiology classification, *n* (%).

Etiology	All (*n* = 39)	Response-Evaluable (*n* = 17)	Non-Evaluable (*n* = 22)
Multifactorial	15 (38.5)	9 (52.9)	6 (27.3)
Inflammatory/chronic disease-associated	9 (23.1)	1 (5.9)	8 (36.4)
CKD-associated	7 (17.9)	5 (29.4)	2 (9.1)
Blood loss-associated	3 (7.7)	0	3 (13.6)
Neoplasia-associated	2 (5.1)	2 (11.8)	0
Critical illness-associated	2 (5.1)	0	2 (9.1)
Other/undetermined	1 (2.6)	0	1 (4.5)

**Table 5:** Response-evaluable cats (*n* = 17): baseline and qualifying HCT, concurrent treatment and response classification.

Case	Etiology	CKD	Cortico-Steroid	DPO Before Qualifying CBC (Day)	Baseline HCT (%)	Qualifying CBC (Day)	Qualifying HCT (%)	ΔHCT (pp)	Last Dose to Qualifying CBC (Days)	Primary Classification	Attribution-Uncertain
1	CKD-associated	Y	Y	Y (0)	39.9	27	29.1	−10.8	≤0	Non-responder ^a^	—
2	Multifactorial	Y	N	N	20.1	24	18.4	−1.7	≤0	Non-responder	—
3	Multifactorial	Y	N	N	21.7	31	30.7	+9.0	24	Responder	Yes (A)
4	CKD-associated	Y	N	Y (14)	22.5	35	39.7	+17.2	22	Responder	Yes (A, B)
5	Multifactorial	Y	N	N	26.0	22 ^b^	27.7	+1.7	2	Non-responder	—
6	CKD-associated	Y	N	N	26.2	28	38.1	+11.9	≤0	Responder	No
7	Multifactorial	Y	N	Y (0, 8, 16)	16.1	30	27.8	+11.7	22	Responder	Yes (A, B)
8	Multifactorial	Y	N	Y (7)	23.1 ^c^	29	17.2	−5.9	≤0	Non-responder	—
9	CKD-associated	Y	N	N	25.3	21	38.5	+13.2	≤0	Responder	No
10	CKD-associated	Y	Y	N	21.3	29	32.9	+11.6	2	Responder	No
11	Multifactorial	N	Y	Y (0, 7)	13.6 ^d^	26	31.6	+18.0	13	Responder	Yes (A, B)
12	Neoplasia-associated	N	Y	N	26.9	22	30.7	+3.8	≤0	Non-responder	—
13	Neoplasia-associated	Y	Y	Y (5, 13)	20.8	33	28.3	+7.5	14	Responder	Yes (A, B)
14	Multifactorial	N	Y	N	22.3 ^d^	36	33.4	+11.1	≤0	Responder	No
15	Multifactorial	N	Y	N	26.5	21	35.1	+8.6	9	Responder	Yes (A)
16	Inflammatory/chronic disease-associated	N	N	N	16.8 ^d^	27	30.9	+14.1	≤0	Responder	No
17	Multifactorial	N	Y	Y (0)	9.6 ^e^	16	28.3	+18.7	15	Responder	Yes (A, B)

CKD, chronic kidney disease; DPO, darbepoetin alfa; HCT, hematocrit; pp, percentage points; ≤0, qualifying CBC obtained on or before the last dose. Corticosteroid: administered before the qualifying CBC. Attribution-uncertainty (post hoc; assessed for responders only): (A) qualifying CBC > 7 days after the last molidustat dose; (B) darbepoetin, transfusion to treat anemia or another intervention expected to affect erythropoiesis between molidustat initiation and the qualifying CBC. ^a^ Also received a transfusion to treat anemia within the response window (day 14). ^b^ Equidistant from day 28 with a CBC on day 34 (HCT 40.3%); the earlier value was used (S3 uses the later value). ^c^ Baseline CBC performed at the referring clinic. ^d^ Transfusion before the baseline CBC; pre-transfusion baseline used in S2. ^e^ Two CBCs on day 0; the value before transfusion was used.

**Table 6:** Proportion of response-evaluable cats meeting the response criterion under the primary analysis, sensitivity analyses specified before formal analysis and post hoc analyses.

Analysis	Status	Description	Responders/*n*	% (95% CI)
Primary	Specified before formal analysis ^b^	As defined in Section 2.4 (≥4 pp); earlier of two equidistant CBCs	12/17	70.6 (46.9–86.7)
S1	Specified before formal analysis ^a^	Excluding cats receiving RRT at initiation (*n* = 1)	12/16	75.0 (50.5–89.8)
S2	Specified before formal analysis	Baseline = last value before transfusion or other acute intervention (3 cats affected)	12/17	70.6 (46.9–86.7)
S1b	Post hoc	Excluding cats receiving RRT or whole-blood transfusion between day −2 and day 2 (*n* = 5)	8/12	66.7 (39.1–86.2)
S3	Post hoc	Later of two equidistant CBCs (1 cat affected)	13/17	76.5 (52.7–90.4)
S4	Post hoc	Attribution-uncertain responders (*n* = 7) reclassified as non-responders	5/17	29.4 (13.3–53.1)
S5	Post hoc	Attribution-uncertain responders (*n* = 7) excluded	5/10	50.0 (23.7–76.3)
S6a	Post hoc	Response threshold ≥ 3 pp	13/17	76.5 (52.7–90.4)
S6b	Post hoc	Response threshold ≥ 5 pp	12/17	70.6 (46.9–86.7)

Proportions refer to response-evaluable cats only and do not estimate the proportion of all treated cats meeting the response criterion. 95% CI, Wilson score interval; pp, percentage points; RRT, extracorporeal renal replacement therapy (intermittent hemodialysis or prolonged intermittent renal replacement therapy). No analysis is designated as the preferred estimate. ^a^ S1, as specified before formal analysis, also listed large-volume transfusion; because whole-blood volume per transfusion was limited by single-donor collection, a volume-based definition could not be applied (Section 2.5). ^b^ The rule for two equidistant CBCs was specified after data review and applied to one cat (Section 2.4).

**Table 7:** Exploratory comparisons of responder status among response-evaluable cats (*n* = 17).

Variable	Exposed: Responders/*n* (%)	Not Exposed: Responders/*n* (%)	OR (95% CI)	*p*
Darbepoetin before qualifying CBC	5/7 (71.4)	7/10 (70.0)	1.07 (0.08–17.40)	1.00
CKD present	7/11 (63.6)	5/6 (83.3)	0.37 (0.01–5.52)	0.60
Corticosteroid before qualifying CBC	6/8 (75.0)	6/9 (66.7)	1.46 (0.12–23.76)	1.00

OR, conditional maximum-likelihood odds ratio with exact 95% CI; *p*, Fisher’s exact test. Analyses are exploratory and were not powered to detect associations. No data were missing for these variables.

**Table 8:** Comparison of the present cohort with the pivotal randomized controlled trial of molidustat in cats.

Feature	Schmidt et al., 2026 [10]	Present Cohort
Design	Multicenter, randomized, double-masked, placebo-controlled	Single-center, retrospective, descriptive
Population	CKD-associated anemia (PCV < 28%)	Heterogeneous etiology (seven categories); 6/39 within the approved indication
Cats assessed for response	75 of 95 randomized (40 molidustat, 35 control)	17 of 39 (response-evaluable)
Main exclusions	Previous ESA, blood transfusion or anabolic steroid treatment; severe inflammation unrelated to CKD	None; concurrent treatments recorded as covariates
Molidustat exposure	5 mg/kg PO SID for 28 consecutive days	5 mg/kg PO SID; median treatment span 22 days (range 2–163) in response-evaluable cats; short and interrupted courses common
Concurrent darbepoetin	Not permitted (ESAs prohibited)	7/17 before the qualifying CBC
Response definition	HCT increase of ≥4 pp or ≥25% from baseline, or both, on day 28	≥4 pp HCT increase at the CBC closest to day 28 (days 14–45), without transfusion to treat anemia in the window
Result	68% vs. 17% (placebo) on day 28	12/17 (70.6%) primary; 29.4–76.5% across sensitivity analyses

ESA, erythropoiesis-stimulating agent; pp, percentage points; PO, orally; SID, once daily.

## Data Availability

The data supporting the findings of this study are available from the corresponding author upon reasonable request. The data are not publicly available due to privacy concerns regarding client-owned animal medical records.

## Supplementary Materials

The following supporting information can be downloaded at: https://www.mdpi.com/article/10.3390/vetsci13101075/s1, Table S1: Operational criteria for the primary anemia etiology categories and conditions recorded in the 39 cats of the descriptive population.

## Abbreviations

The following abbreviations are used in this manuscript:

CBC	complete blood count
CKD	chronic kidney disease
DPO	darbepoetin alfa
ESA	erythropoiesis-stimulating agent
Hb	Hemoglobin
HCT	Hematocrit
HIF-PHI	hypoxia-inducible factor prolyl hydroxylase inhibitor
IRIS	International Renal Interest Society
PCV	packed cell volume
PIRRT	prolonged intermittent renal replacement therapy
RRT	extracorporeal renal replacement therapy
pp	percentage points
