# Supplementary material for: Hematologic Responses to Molidustat in Cats with Anemia of Heterogeneous Etiology: A Retrospective Descriptive Study
Source: Vet Sci. 2026 Oct 11;13(10):1075. doi: 10.3390/vetsci13101075 (PMC13709430; doi:10.3390/vetsci13101075)
Supplement: Supplementary file 1 [file vetsci-13-01075-s001.zip › vetsci-4595963-supplementary.pdf]

Supplementary Material

Hematologic Responses to Molidustat in Cats with Anemia of Heterogeneous Etiology: A Retrospective Descriptive Study

Table S1. Operational criteria for the primary anemia etiology categories and conditions recorded in the 39 cats of the descriptive population.

Category	Operational criterion	n (all / response-evaluable)	Conditions recorded in this cohort
CKD-associated	CKD (IRIS criteria) or acute-on-chronic kidney disease judged to be the principal cause of anemia; other conditions, if present, judged not to contribute comparably	7 / 5	CKD (IRIS stage 2–4 by creatinine at presentation); acute-on-chronic kidney disease, including cases with ureteral obstruction and cases managed with renal replacement therapy; concurrent conditions (e.g., otitis media, nephrolithiasis) recorded
Inflammatory/chronic disease-associated	Infectious, inflammatory or other chronic systemic disease judged to be the principal cause, without an immune-mediated component and without CKD judged to contribute comparably	9 / 1	Feline infectious peritonitis; feline panleukopenia (with or without calicivirus infection); hepatic lipidosis; pyonephritis
Neoplasia-associated	Neoplasia confirmed by histopathology and immunohistochemistry (after splenectomy or intestinal biopsy) and judged to be the principal cause	2 / 2	Intestinal lymphoma; splenic mast cell tumor with suspected metastasis
Blood loss-associated	Documented hemorrhage (clinical, endoscopic or imaging evidence) judged to be the principal cause	3 / 0	Acute gastric hemorrhage; acute hemoabdomen; oral hemorrhage associated with severe periodontitis and mandibular/alveolar bone lysis
Critical illness-associated	Anemia developing during an acute critical illness in the absence of a single identified mechanism, with no pre-existing condition judged to be the principal cause	2 / 0	Acute kidney injury managed with renal replacement therapy; acute neurological disease (seizures) with acute kidney injury
Multifactorial	Two or more conditions judged to contribute comparably; all cats with immune-mediated anemia (diagnosed or presumptive) were assigned to this category and the immune-mediated component and its diagnostic basis were recorded	15 / 9	CKD with concurrent neoplasia (nasal lymphoma, mast cell tumor, hepatic tumor), hepatobiliary or pancreatic disease, cardiomyopathy, hypertensive target-organ damage or systemic inflammation; immune-mediated anemia with FeLV infection, osteomyelitis/myelofibrosis or cholangiohepatitis; small cell lymphoma with hyperthyroidism

Other/undetermined	No cause identified after clinical evaluation	1 / 0	Nonregenerative pancytopenia of undetermined cause

Etiology was assigned from the medical record by the attending clinician-investigator; where more than one diagnosis plausibly contributed and no single cause predominated, the case was classified as multifactorial. CKD status, regenerative status and concurrent treatments were recorded as separate covariates (Section 2.2). Diagnoses are listed at the level of detail documented in the medical record. CKD, chronic kidney disease; FeLV, feline leukemia virus; IRIS, International Renal Interest Society.
